# A U‐shaped relationship between left ventricular ejection fraction and risk of worsening heart failure

**DOI:** 10.1002/ejhf.70061

**Published:** 2025-10-20

**Authors:** Hao‐Chih Chang, Wei‐Ming Huang, Liang‐Yin Lin, Ching‐Wei Lee, Chih‐Hsueh Tseng, Wen‐Chung Yu, Hao‐Min Cheng, Chern‐En Chiang, Chen‐Huan Chen, Shih‐Hsien Sung

**Affiliations:** ^1^ Cardiovascular Center Taipei Veterans General Hospital Taipei Taiwan; ^2^ Department of Internal Medicine College of Medicine, National Yang Ming Chiao Tung University Taipei Taiwan; ^3^ Institute of Public Health, College of Medicine, National Yang Ming Chiao Tung University Taipei Taiwan; ^4^ Institute of Emergency and Critical Care Medicine, College of Medicine, National Yang Ming Chiao Tung University Taipei Taiwan; ^5^ Division of Cardiology, Department of Internal Medicine E‐Da Hospital, I‐Shou University Kaohsiung Taiwan; ^6^ Division of Faculty Development, Department of Medical Education Taipei Veterans General Hospital Taipei Taiwan; ^7^ General Clinical Research Center Taipei Veterans General Hospital Taipei Taiwan

**Keywords:** Left ventricular ejection fraction, Worsening heart failure, Supranormal ejection fraction

## Abstract

**Aims:**

Left ventricular ejection fraction (LVEF) is a key measure of cardiac function. While prior studies showed a U‐shaped relationship between LVEF and mortality, its association with worsening heart failure (HF) remains unclear. We aimed to evaluate the association between the full spectrum of LVEF and the risk of worsening HF.

**Methods and results:**

We analysed data from 93 694 consecutive participants (median age 62 years [interquartile range: 50–76 years], 51.4% men) undergoing echocardiography at a tertiary medical centre. LVEF, measured by biplane Simpson's method, was categorized into 5% intervals from <20% to ≥70%. The primary outcome was a composite of all‐cause mortality or worsening HF, while the secondary outcomes included all‐cause mortality, cardiovascular death, and worsening HF. The primary outcome occurred in 32 398 (34.6%) participants over a median follow‐up of 8.3 years. A U‐shaped relationship between LVEF and the primary outcome was observed, with a nadir at 60–70% and an increased risk when LVEF was ≥70% [adjusted hazard ratio (aHR) 1.12; 95% confidence interval (CI) 1.06–1.18]. Similar patterns were observed for the secondary outcomes. Participants with LVEF ≥70% also had a higher risk of worsening HF (aHR 1.13, 95% CI 1.03–1.23). This U‐shaped association was consistent across subgroups stratified by age, sex, hypertension, and diabetes, and was observed for both incident and recurrent HF events.

**Conclusions:**

Left ventricular ejection fraction demonstrated a U‐shaped association with worsening HF, with the lowest risk at 60–70%. Supranormal LVEF (≥70%) identified a high‐risk phenotype, underscoring the need for tailored management strategies for this subgroup.

## Introduction

Left ventricular ejection fraction (LVEF) is a commonly used imaging measure for assessing systolic function of the left ventricle and is fundamental in the clinical management of heart failure (HF). As a primary metric, LVEF aids in patient phenotyping, guides HF‐specific treatment, and carries significant prognostic implications across various patient populations.^1^, ^2^, ^3^ While its predictive value for mortality has been well‐established in clinical trials and community registries,^4^, ^5^, ^6^ a U‐shaped relationship between the spectrum of LVEF and mortality risk has been observed.^4^ This U‐shaped relationship defines a novel phenotype of supranormal LVEF, which carries a mortality risk similar to reduced LVEF.

However, the correlation between LVEF and the incidence of worsening HF, particularly over the full spectrum of LVEF values, remains insufficiently explored. Previous studies have predominantly concentrated on patients with established HF,^7^, ^8^ often categorizing LVEF into limited ranges^7^, ^8^, ^9^ or focusing primarily on elderly populations.^10^ This segmentation of LVEF and demographic focus have left a gap in understanding how LVEF extremes impact HF incidence in a broader, more diverse population. Given the emerging evidence that sodium–glucose co‐transporter 2 (SGLT2) inhibitors reduce the risk of incident HF,^11^, ^12^, ^13^ a holistic evaluation of the association between the spectrum of LVEF and HF risk in clinical practice is necessary. Therefore, this study aimed to investigate the association between the continuum of LVEF and the risk of worsening HF, employing a large‐scale, hospital‐based cohort.

## Methods

### Study design and study population

We conducted a retrospective cohort study using the echocardiography database in a tertiary medical centre in Taiwan between April 2005 and December 2017. We identified consecutive outpatients undergoing their first echocardiography for a broad range of indications, including dyspnoea, chest pain, suspected HF, pre‐operative evaluations, or general health evaluation. Patients with significant valvular heart disease, defined as severe mitral regurgitation, mitral stenosis, aortic regurgitation, or aortic stenosis, were excluded, as such conditions may alter the prognostic relevance of LVEF.^14^, ^15^, ^16^, ^17^ Data on demographic characteristics, body mass index (BMI), systolic blood pressure, heart rate, and laboratory results were collected. We linked our institutional database to Taiwan's National Health Insurance Research Database (NHIRD), a nationwide, single‐payer, compulsory health insurance system that provides comprehensive coverage for more than 99% of the country's population. Comorbidities were identified using the diagnostic codes from the International Classification of Diseases, Ninth or Tenth Revision, Clinical Modification (ICD‐9‐CM or ICD‐10‐CM) (online supplementary *Methods*). Medications prescribed within 2 months before and 4 months after the index echocardiography were identified using the Anatomical Therapeutic Chemical classification system. The investigation conformed to the principles outlined in the Declaration of Helsinki. The Institutional Review Committee of Taipei Veterans General Hospital approved the use of registry data for research purposes and waived the requirement for informed consent.

### Measurement of left ventricular ejection fraction

The transthoracic echocardiographic study was conducted according to the recommendations of the American Society of Echocardiography.^18^ LVEF was calculated from the left ventricular end‐diastolic volume and end‐systolic volume estimates using the biplane Simpson's method. All LVEFs were categorized into 5% intervals, consistent with accepted thresholds for interobserver variability and measurement disagreement,^19^, ^20^, ^21^ with the lowest intervals <20% and the highest intervals ≥70%. Left atrial dimension, interventricular septum (IVS) thickness, posterior wall thickness (PWT), and left ventricular internal diameter at end‐diastole (LVIDd) were measured using M‐mode echocardiography. Additional Doppler parameters, including early diastolic transmitral flow velocity (E), septal mitral annular tissue velocity (e'), and peak tricuspid regurgitation velocity (TR Vmax), were also recorded. All the echocardiographic images were reviewed by board‐certified echocardiographers.

### Study outcomes

The primary outcome was a composite of all‐cause mortality or worsening HF, defined as either hospitalization for HF or an urgent visit to the emergency department resulting in intravenous diuretics for HF.^22^, ^23^ Secondary outcomes included all‐cause mortality, cardiovascular death, and worsening HF. For all‐cause mortality and cardiovascular death, we linked to the National Death Registry, an official and obligatory death registration system in Taiwan, which records the date and causes of death according to the ICD codes for all citizens. Deaths linked to ICD‐9 codes 390–448 or ICD‐10 codes I00–I99 were classified as cardiovascular deaths. HF hospitalization was identified through linkage with Taiwan's NHIRD, which provides comprehensive records of both primary and secondary diagnoses for each admission. Similarly, emergency department visits resulting in the administration of intravenous loop diuretics for HF were also captured through the same database.

### Statistical analysis

Baseline characteristics were described as median ± interquartile range (IQR) for continuous variables and percentages for categorical variables. Comparisons of continuous variables were performed using the Mann–Whitney U or Kruskal–Wallis test, as appropriate, whereas categorical variables were compared using the chi‐squared test. Missing data were addressed using multiple imputation via fully conditional specification with 20 imputations performed. For primary analysis, we included each patient's first echocardiography, with follow‐up defined as the time from the index echocardiography to the occurrence of study outcomes or the administrative censoring date of 31 December 2022. The association between each group of LVEF (vs. reference group: LVEF 60–65%) and the study outcomes was assessed in a time‐to‐first event analysis with the use of multivariable Cox proportional‐hazards regression analysis, accounting for age, sex, BMI, comorbidities, concurrent use of renin–angiotensin system (RAS) inhibitors, β‐blockers, mineralocorticoid receptor antagonists (MRA), and SGLT2 inhibitors, year of echocardiogram, heart rate, LVIDd, left ventricular hypertrophy (LVH), and measures of diastolic function, including left atrial size, E wave velocity, e' velocity, and TR Vmax. Adjusted hazard ratios (aHR), 95% confidence intervals (CI), and *p*‐values were reported. Sensitivity analyses were conducted: (i) to incorporate all echocardiograms performed during follow‐up and treat LVEF as a time‐varying covariate in Cox proportional hazards models; (ii) to evaluate the risk of HF hospitalization as the sole HF outcome across the LVEF spectrum; (iii) to assess the risk of incident HF by excluding subjects with a prior history of HF or baseline use of oral loop diuretics; (iv) to analyse recurrent HF hospitalizations using the counting‐process formulation of the Cox proportional hazards model^24^; and (v) to adjust for the clinical indications of echocardiography in the multivariable models. Additional analyses examined interactions between LVEF and key subgroups, including age, sex, hypertension, and diabetes status. All statistical analyses were performed using SAS version 9.4 (IBM, Cary, NC, USA) and R software version 4.0.2 (R Foundation for Statistical Computing, Vienna, Austria). All tests were two‐sided, and *p* < 0.05 was considered statistically significant.

## Results

### Study population

**Figure 1:**
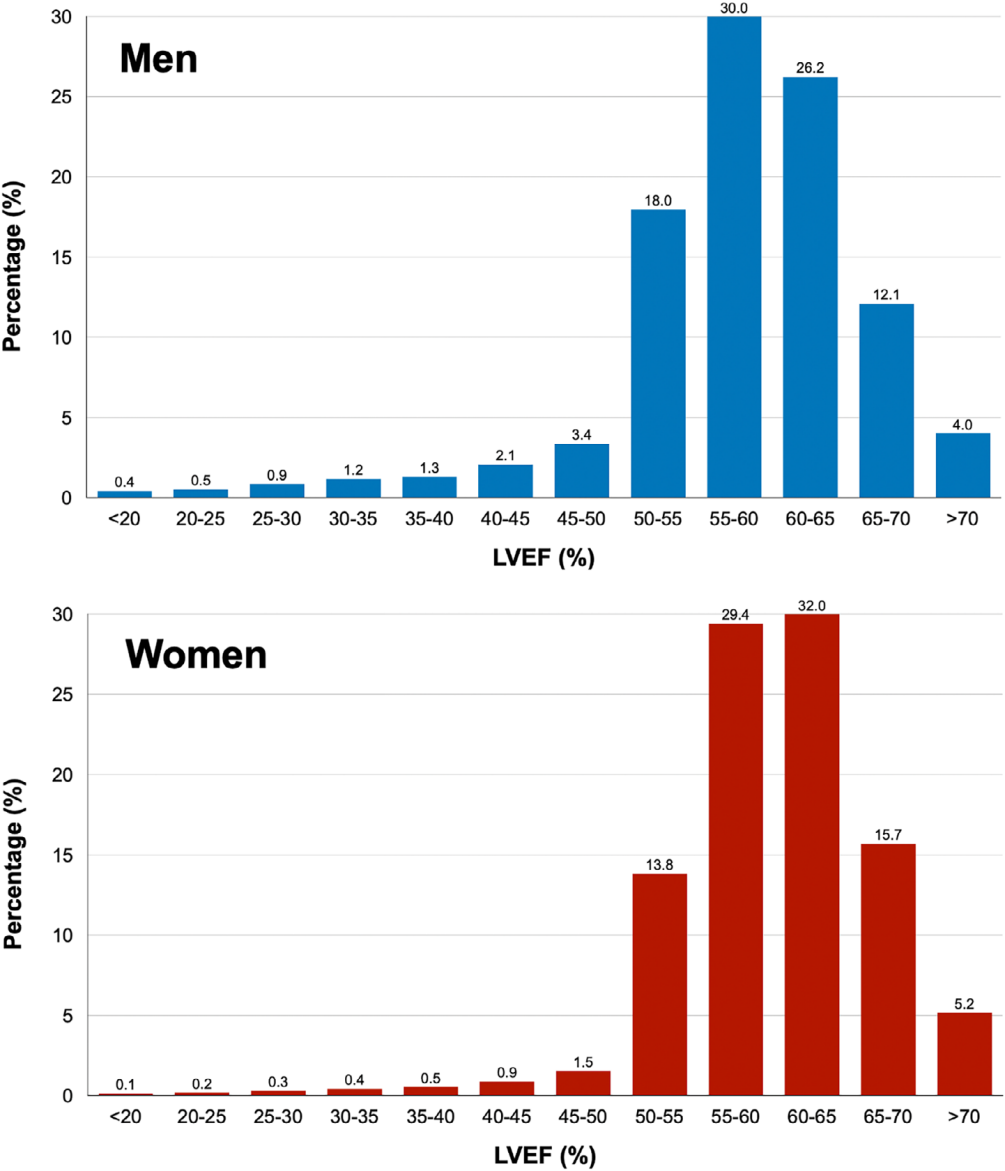
The distribution of left ventricular ejection fraction (LVEF) in the study population stratified by sex. Women were more prevalent than men in the LVEF subgroups above 60%, while men were more prevalent than women in the LVEF subgroups below 55%.

This study included 93 694 consecutive participants (median age 62 years [IQR: 50–76 years], 51.4% men). *Figure* *1* illustrates the distribution of LVEF across genders. The most frequently reported LVEF category was 55–60% in men (30%) and 60–65% in women (32%). The leading indications for echocardiography were dyspnoea (37.3%), suspected HF (33%), and chest pain (18.2%).

*Table* *1* shows the baseline characteristics stratified by LVEF subgroups in men and women, revealing significant heterogeneity between subgroups (all *p*‐values <0.001, except for the use of SGLT2 inhibitors). Compared with the group of LVEF 60–70%, individuals who deviated from this range were older and had a higher prevalence of hypertension, diabetes, hyperlipidaemia, atrial fibrillation, coronary artery disease, and a history of HF. They also had lower haemoglobin levels, lower estimated glomerular filtration rate, higher natriuretic peptide levels, larger left atrial size, larger LVIDd, more LVH, and higher TR Vmax. Individuals with lower LVEF were more often prescribed RAS inhibitors, β‐blockers, MRAs, loop diuretics, digoxin, and statins, whereas those with LVEF >70% were more likely to receive RAS inhibitors, loop diuretics, and digoxin.

**Table 1:** Baseline characteristics of the study population

Variables	Men					Women				
	LVEF ≤40% (*n* = 2206)	40% < LVEF <50% (*n* = 2625)	50% ≤ LVEF <60% (*n* = 23 115)	LVEF 60–70% (*n* = 18 360)	LVEF >70% (*n* = 1875)	LVEF ≤40% (*n* = 694)	40% < LVEF <50% (*n* = 1081)	50% ≤ LVEF <60% (*n* = 19 731)	LVEF 60–70% (*n* = 21 690)	LVEF >70% (*n* = 2317)
**Demographics**										
Age, years	73 (58–83)*	74 (59–83)*	64 (51–80)*	63 (51–78)	68 (54–80)*	72 (61–80)*	71 (59–81)*	60 (47–72)*	59 (47–70)	63 (52–74)*
BMI, kg/m^2^	23.6 (21.1–26.4)*	24.3 (21.6–26.8)*	24.7 (22.3–27.2)*	24.6 (22.4–26.9)	24.9 (22.6–27.2)*	23.3 (20.3–26.5)*	23.6 (20.9–27.1)	24 (21.4–27.1)*	23.8 (21.2–26.7)	24.1 (21.6–27.1)*
SBP, mmHg	123 (110–137)*	129 (116–142)*	129 (117–142)	130 (118–142)	130 (120–143)*	126 (111141)	129.5 (114–145)*	127 (114–140.5)*	126 (113–140)	129 (116–143)*
HR, bpm	80 (70–91)*	78 (69–89)*	77 (68–87)*	76 (67–86)	76 (68–87)	84 (74–95)*	81 (71–92)*	78 (70–88)*	78 (69–87)	78 (69–87)
**Comorbidities, *n* (%)**										
Hypertension	1520 (68.9)*	1747 (77.1)*	12 471 (54)*	9392 (51.2)	1111 (59.3)*	453 (65.3)*	680 (62.9)*	8317 (42.2)*	8432 (38.9)	1117 (48.2)*
Diabetes	841 (38.1)*	868 (38.3)*	5237 (22.7)*	3690 (20.1)	411 (21.9)*	281 (40.5)*	346 (32)*	3565 (18.1)*	3460 (16)	467 (20.2)*
Hyperlipidaemia	686 (31.1)*	815 (36)*	5901 (25.5)	4446 (24.2)	462 (24.6)	215 (31)*	337 (31.2)*	4683 (23.7)	5091 (23.5)	614 (26.5)*
Atrial fibrillation	423 (19.2)*	448 (19.8)*	2324 (10.1)*	1405 (7.7)	173 (9.2)*	126 (18.2)*	189 (17.5)*	1235 (6.3)*	873 (4)	113 (4.9)*
CAD	1216 (55.1)*	1402 (61.9)*	9326 (40.3)*	6996 (38.1)	807 (43)*	316 (45.5)*	447 (41.4)*	5850 (29.6)*	6031 (27.8)	775 (33.4)*
Heart failure	981 (44.5)*	659 (29.1)*	2210 (9.9)*	1290 (7)	192 (10.2)*	360 (51.9)*	313 (29)*	1706 (8.6)*	1322 (6.1)	167 (7.2)*
**Haemogram and biochemistries**										
Haemoglobin, g/dl	12.1 (10.2–13.8)*	12.2 (10.4–14.1)*	13.4 (11.6–14.8)*	13.5 (11.7–14.8)	13.4 (11.1–14.8)	11.2 (10.1–12.6)*	11.3 (9.8–12.65)*	12.5 (11.2–13.4)*	12.6 (11.4–13.5)	12.4 (11.1–13.4)*
Sodium, mmol/L	139 (137–142)*	140 (137–142)*	140 (138–142)	140 (138–142)	140 (137–141)*	139 (136–141)*	139 (137–141)*	140 (138–142)	141 (138–142)	140 (138–142)*
eGFR, ml/min/1.73 m^2^	60 (36–81)*	68 (44–88)*	79 (58–95)*	82 (60–97)	77 (54–92)*	58 (32–78)*	65 (35–88)*	88 (65–102)*	91 (70–104)	87.9 (64.1–101)*
NT‐proBNP, pg/ml (*n* = 3774 in males and *n* = 2415 in females)	6524 (2752–17 955)*	3205 (1098–9935)*	1080 (241.7–3749)*	694.5 (165.6–2451)	1072.5 (234.8–3735)	8601 (3457–25 215)*	5003.5 (1729–14 938)*	1110.5 (226.1–4823)*	643.4 (120–3313)	1853 (399.8–4867)*
**Echocardiography**										
LA diameter, mm	44.1 (38.9–49.7)*	40.4 (35.8–45.6)*	37.4 (33–42.1)*	37.1 (33.2–41.4)	38.9 (34.6–43)*	42.3 (37.6–46.9)*	39.6 (33.7–45.8)*	34.3 (30.2–39)*	34 (30.4–38)	35.9 (32–40)*
IVS, mm	10.5 (9.1–11.8)*	10.7 (9.4–12)*	10.2 (9–11.5)*	10 (9–11.4)	10.2 (9–12)*	10.2 (9.1–11.7)*	10.2 (8.9–11.5)*	9 (8–10.6)*	9 (8–10.1)	9 (8–10.8)*
PWT, mm	10.5 (9.4–11.7)*	10.6 (9.5–11.7)*	10 (9–11.2)*	10 (9–11)	10 (9–11.3)*	10.2 (9.1–11.3)*	10.25 (8.9–11.3)*	9 (8–10.3)*	9 (8–10)	9 (8–10.3)*
LVIDd, mm	56.7 (51–61.8)*	51.4 (46.5–56)*	48.45 (44.6–52.3)*	48 (44.1–52)	48.8 (44.2–52.65)*	54 (48.8–58.3)*	47.8 (42.95–52.65)*	45 (41.7–48.8)*	45 (41.5–48)	45 (42–49)*
LVH, *n* (%)	283 (12.8)*	331 (14.6)*	1917 (8.3)*	1402 (7.6)	268 (14.3)*	64 (9.2)*	113 (10.5)*	715 (3.6)*	664 (3.1)	155 (6.7)*
LVEF, %	32 (26–36)*	46 (44–48)*	56 (54–58)*	63 (62–66)	73 (71–75)*	33 (27–36)*	46 (44–49)*	57 (54–58)*	63 (62–66)	73 (71–75)*
E, cm/s	86.9 (65.2–106.6)*	74 (57.2–95.8)*	71.1 (58.7–86.9)*	72.6 (60.7–86.9)	71.8 (59.7–88.3)	91.3 (67.9–113)*	83 (63.7–106.1)*	78 (64.7–92.8)*	79.5 (66.6–93)	78 (65–92.9)*
e', cm/s	4.7 (3.7–6)*	5.5 (4.3–6.8)*	6.5 (5.2–8.2)*	6.8 (5.5–8.5)	6.7 (5.4–8.2)	4.3 (3.4–5.6)*	5.3 (4.2–6.8)*	6.6 (5.2–8.8)*	7 (5.4–9.2)	6.6 (5.1–8.4)*
TR Vmax, m/s	2.8 (2.4–3.3)*	2.6 (2.2–2.9)*	2.38 (2.12–2.69)	2.39 (2.12–2.67)	2.41 (2.12–2.71)*	2.9 (2.5–3.3)*	2.6 (2.3–3.1)*	2.35 (2.06–2.64)*	2.34 (2.08–2.61)	2.4 (2.1–2.7)*
**Medications, *n* (%)**										
RAS inhibitors	1457 (66)*	1534 (67.7)*	10 407 (45)*	7836 (42.7)	943 (50.3)*	461 (66.4)*	591 (54.7)*	6669 (33.8)*	6617 (30.5)	851 (36.7)*
β‐blockers	1383 (62.7)*	1384 (61.1)*	9565 (41.4)*	7145 (38.9)	758 (40.4)	452 (65.1)*	599 (55.4)*	9170 (46.5)*	9726 (44.8)	1090 (47)*
MRA	994 (45.1)*	482 (21.3)*	1514 (6.5)*	960 (5.2)	116 (6.2)	349 (50.3)*	228 (21.1)*	1033 (5.2)*	870 (4)	100 (4.3)
SGLT2 inhibitors	6 (0.3)	5 (0.2)	46 (0.2)	24 (0.1)	0 (0)	2 (0.3)	1 (0.1)	19 (0.1)	25 (0.1)	1 (0.0004)
Loop diuretics	1462 (66.3)*	1066 (47.1)*	5202 (22.5)*	3592 (19.6)	508 (27.1)*	510 (73.5)*	476 (44)*	3620 (18.3)*	3184 (14.7)	495 (21.4)*
Digoxin	435 (19.7)*	213 (9.4)*	778 (3.4)*	407 (2.2)	66 (3.5)*	176 (25.4)*	134 (12.4)*	575 (2.9)*	355 (1.6)	66 (2.8)*
Statin	775 (35.1)*	904 (39.9)*	5524 (23.9)*	4170 (22.7)	428 (22.8)	218 (31.4)*	302 (27.9)*	4057 (20.6)	4386 (20.2)	476 (20.5)

BMI, body mass index; CAD, coronary artery disease; E, early diastolic transmitral flow velocity; e', early diastolic mitral annular velocity; eGFR, estimated glomerular filtration rate; HR, heart rate; IVS, interventricular septum; LA, left atrial; LVEF, left ventricular ejection fraction; LVH, left ventricular hypertrophy; LVIDd, left ventricular internal dimension at end‐diastole; MRA, mineralocorticoid receptor antagonist; NT‐proBNP, N‐terminal pro‐B‐type natriuretic peptide; PWT, posterior wall thickness; RAS, renin–angiotensin system; SBP, systolic blood pressure; SGLT2, sodium–glucose co‐transporter 2; TR Vmax, tricuspid regurgitation peak velocity.

*p < 0.05 compared with the reference group of LVEF 60–70% using the Mann–Whitney U test.

### Primary outcome

**Figure 2:**
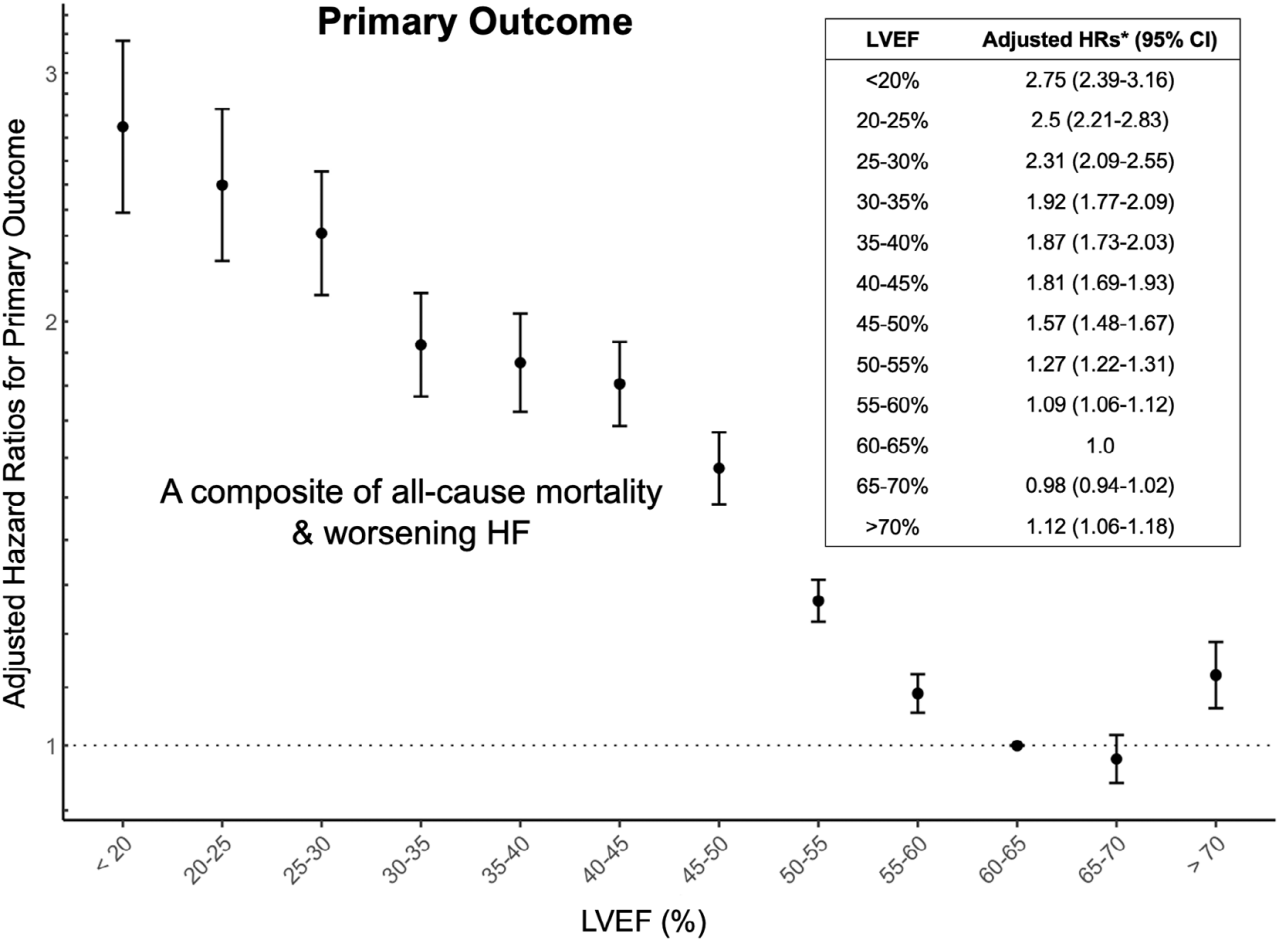
Association between left ventricular ejection fraction (LVEF) and the primary outcome. A U‐shaped relationship can be observed between the spectrum of LVEF and the risk of the primary outcome, a composite of all‐cause mortality or worsening heart failure (HF). CI, confidence interval; HR, hazard ratio. *Adjusting for age, sex, body mass index, comorbidities, renin–angiotensin system inhibitors, β‐blockers, mineralocorticoid receptor antagonists, sodium–glucose co‐transporter 2 inhibitors, year of echocardiogram, heart rate, left ventricular internal diameter at end‐diastole, left ventricular hypertrophy, left atrial size, E wave velocity, e' velocity, and tricuspid regurgitation peak velocity.

During a median follow‐up of 8.3 years (IQR: 5.3–11.2 years), the primary outcome occurred in 32 398 (34.6%) participants. Adjusted HRs revealed a U‐shaped relationship between LVEF and the primary outcome, which comprised all‐cause mortality or worsening HF, with a nadir of risk at LVEF of 60–70% (*Figure* *2*). Outside this range, the risk increased as LVEF deviated either above or below this interval. Specifically, the adjusted HR was significantly elevated for LVEF ≥70% (aHR 1.12; 95% CI 1.06–1.18) and for LVEF of 55–60% (aHR 1.09; 95% CI 1.06–1.12).

### Secondary outcomes

**Figure 3:**
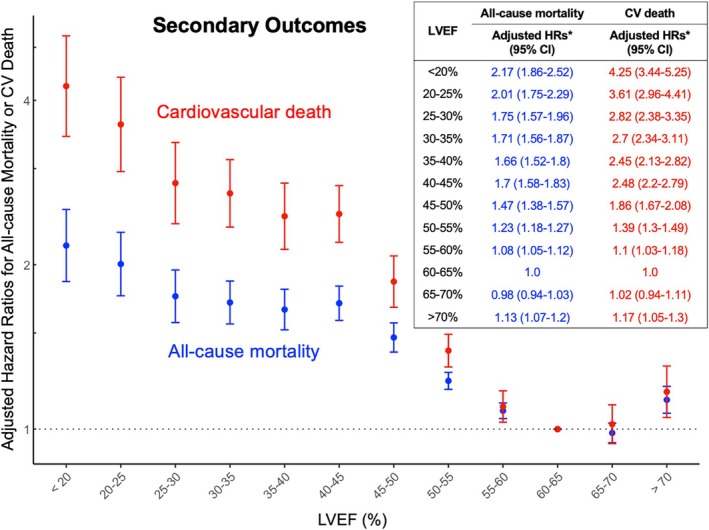
Association between left ventricular ejection fraction (LVEF) and the secondary outcomes: all‐cause mortality and cardiovascular (CV) death. A similar U‐shaped relationship is shown between LVEF and the risk of all‐cause mortality and CV death. Participants with LVEF ≥70% exhibited a significantly higher risk of both all‐cause mortality and CV death when compared with the reference group of LVEF 60–65%. CI, confidence interval; HR, hazard ratio. *Adjusting for age, sex, body mass index, comorbidities, renin–angiotensin system inhibitors, β‐blockers, mineralocorticoid receptor antagonists, sodium–glucose co‐transporter inhibitors, year of echocardiogram, heart rate, left ventricular internal diameter at end‐diastole, left ventricular hypertrophy, left atrial size, E wave velocity, e' velocity, and tricuspid regurgitation peak velocity.

**Figure 4:**
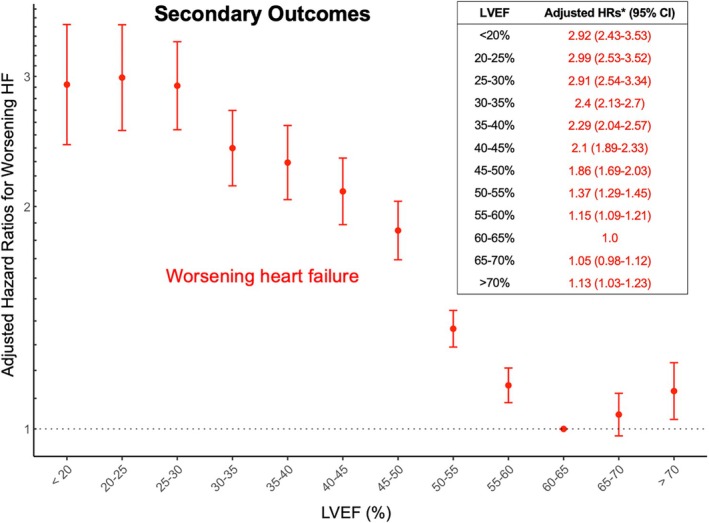
Association between left ventricular ejection fraction (LVEF) and the secondary outcomes: worsening heart failure (HF). The U‐shaped relationship between LVEF and the risk of worsening HF remained evident, underscoring LVEF ≥70% as a high‐risk phenotype for HF. CI, confidence interval; HR, hazard ratio. *Adjusting for age, sex, body mass index, comorbidities, renin–angiotensin system inhibitors, β‐blockers, mineralocorticoid receptor antagonists, sodium–glucose co‐transporter inhibitors, year of echocardiogram, heart rate, left ventricular internal diameter at end‐diastole, left ventricular hypertrophy, left atrial size, E wave velocity, e' velocity, and tricuspid regurgitation peak velocity.

For secondary outcomes, 29 286 participants (31.3%) died during follow‐up, including 8300 (8.9%) cardiovascular deaths. Additionally, 12 204 participants (13%) experienced worsening HF. *Figures* *3* and *4* show the fully adjusted HRs for all‐cause mortality, cardiovascular death, and worsening HF, respectively. A similar U‐shaped relationship was observed between LVEF and each outcome, with the nadir risk at LVEF of 60–70%. Notably, the risk gradient was steeper for cardiovascular death and worsening HF than for all‐cause mortality. Individuals with an LVEF ≥70% had a significantly increased risk of cardiovascular death (aHR 1.17; 95% CI 1.05–1.30) and worsening HF (aHR 1.13; 95% CI 1.03–1.23).

### Sensitivity analyses

When all follow‐up echocardiograms were included, a total of 155 245 echocardiograms were recorded for the 93 694 study participants. The adjusted HRs for LVEF as a time‐varying variable continued to demonstrate a U‐shaped relationship with the primary outcome (online supplementary *Figure* *S1*). The lowest risk remained at an LVEF of 60–70%, while individuals with an LVEF ≥70% were associated with a significantly increased risk for the primary outcome (aHR 1.15; 95% CI 1.09–1.22). Similar patterns were observed for the secondary outcomes (online supplementary *Figure* *S2*).

A similar U‐shaped association was observed for HF hospitalization alone, with a nadir at LVEF of 60–70% and an increased risk at LVEF ≥70% (aHR 1.17; 95% CI 1.01–1.35) (online supplementary *Figure* *S3*). After excluding patients with a history of HF (*n* = 9281) or baseline use of oral loop diuretics (*n* = 20 115), the U‐shaped relationship between LVEF and the risk of incident HF persisted, with LVEF ≥70% still associated with increased risk (online supplementary *Figures* *S4* and *S5*).

Recurrent event analysis also demonstrated a similar U‐shaped association between LVEF and the risk of recurrent HF hospitalization (online supplementary *Figure* *S6*). Adjustments for the indications of echocardiography yielded similar results for both primary (online supplementary *Figure* *S7*) and secondary outcomes (online supplementary *Figure* *S8*).

### Interactions between age, sex, comorbidities, and left ventricular ejection fraction

The relationship between LVEF and the risk of worsening HF remained U‐shaped across all age groups and in both sexes, with the lowest risk observed at an LVEF of 60–70% (online supplementary *Figure* *S9*). Similar patterns were seen regardless of the presence or absence of hypertension or diabetes (online supplementary *Figure* *S10*). Significant interactions were observed between LVEF and age (*p* < 0.001), sex (*p* = 0.004), hypertension (*p* < 0.001), and diabetes (*p* < 0.001), indicating a significantly higher risk of worsening HF among older individuals, men, and those with hypertension or diabetes.

## Discussion

In this exploratory study, we identified a U‐shaped relationship between LVEF and the risk of the primary outcome, cardiovascular death, and worsening HF (*Graphical Abstract*), with the lowest risk at an LVEF of 60–70%. Notably, the association with worsening HF showed a steeper risk gradient outside this optimal range than all‐cause mortality, suggesting greater sensitivity to LVEF variations. Similar U‐shaped patterns for both incident HF and recurrent HF events further support these findings.

### Left ventricular ejection fraction‐centric view in heart failure

Currently, LVEF remains the principal measure for HF classification and management.^25^ Traditionally, HF has been categorized by LVEF into reduced (≤40%), mildly reduced (41–49%), and preserved (≥50%).^25^ However, recent clinical trials of SGLT2 inhibitors^26^, ^27^ and pooled analyses^28^, ^29^, ^30^ have challenged this framework. Evidence suggests that the benefits of HF therapies extend well beyond the conventional cutoff of 50%.^29^, ^30^, ^31^, ^32^ A new taxonomy substituting ‘HF with preserved LVEF’ with ‘HF with normal LVEF’ is emerging.^25^, ^33^ Despite these developments, the association between the entire spectrum of LVEF and HF risk has not been fully explored. Our study addressed this gap and supported the use of ≥60% as a more appropriate threshold for defining ‘normal’ LVEF. Importantly, the paradoxically increased risk in individuals with LVEF ≥70% calls for re‐evaluating the upper limit of ‘normal’ LVEF and highlights that supranormal LVEF can represent a distinct high‐risk phenotype.

### Supranormal left ventricular ejection fraction and the risk of heart failure

Since the study by Wehner *et al*.,^4^ supranormal LVEF has garnered increasing attention as a potential high‐risk phenotype. However, subsequent studies reported inconsistent findings.^5^, ^34^, ^35^, ^36^, ^37^, ^38^ Most of these studies either focused exclusively on patients with established HF^35^, ^36^, ^37^, ^38^ or did not specifically assess the risk of incident or worsening HF.^5^ In contrast, our study encompassed a broader and more heterogeneous population, and uniquely evaluated worsening HF, including both hospitalizations and urgent outpatient events, as a primary outcome. The observed increased risk with supranormal LVEF (≥70%) underscores a higher‐risk HF phenotype within the heterogeneous HF with preserved ejection fraction spectrum. While pharmacologic therapies showed efficacy across preserved LVEF values,^39^ their therapeutic benefits diminished when LVEF was above 65%.^29^, ^30^, ^31^ Due to limited representation of individuals with supranormal LVEF in major clinical trials,^26^, ^27^, ^40^ evidence for managing this subgroup remains insufficient. Our findings underscore a mismatch between the elevated HF risk in patients with supranormal LVEF and the lack of evidence‐based therapies. Recognizing this subgroup is essential, as they may benefit from closer monitoring and further evaluation for underlying conditions, such as hypertrophic remodelling, restrictive physiology, or infiltrative diseases.^41^

### Study limitations

This study was subject to several limitations. First, we did not use three‐dimensional echocardiography or cardiac magnetic resonance imaging to assess LVEF. To mitigate measurement variability, we employed the biplane Simpson's method for LVEF quantification as recommended by the American Society of Echocardiography^18^ and categorized LVEF into 5% intervals to account for interobserver variability.^20^, ^21^ Second, although this study utilized detailed clinical data from a single‐centre administrative database, which enhanced internal validity, generalizability to other healthcare settings or populations may be limited. However, by linking to the NHIRD, we minimized selection bias and ensured near‐complete follow‐up. Third, due to the observational study design, residual confounding and unobserved biases may still be present, even though we have adjusted for all potential confounders measured in the registry.

## Conclusions

This study demonstrated a U‐shaped relationship between LVEF and the risks of all‐cause mortality, cardiovascular death, and worsening HF, with the nadir of risk at an LVEF of 60–70%. While reaffirming the known risks of reduced LVEF, our study underscored the underrecognized HF risk of patients with supranormal LVEF. These findings support a more nuanced approach to HF management, emphasizing the need for comprehensive risk assessment and personalized treatment strategies across the full LVEF spectrum.

### Funding

This work received support from Ministry of Health and Welfare, Taiwan (grant numbers: MOHW113‐TDU‐B‐211‐114007, MOHW114‐TDU‐B‐211‐124002), National Science and Technology Council, Taiwan (grant numbers: NSTC111‐2314‐B‐A49‐010, NSTC112‐2314‐B‐A49‐042, NSTC113‐2314‐B‐A49‐041), Taipei Veterans General Hospital‐National Taiwan University Hospital Joint Research Program (grant numbers: VN111‐04, VN112‐05) and Taipei Veterans General Hospital (grant numbers: V107C‐027, V108‐133).

**Conflict of interest**: none declared.

## Supporting information


**Appendix S1.** Supporting Information.
