# Supplementary material for: A U‐shaped relationship between left ventricular ejection fraction and risk of worsening heart failure
Source: Eur J Heart Fail. 2025 Oct 20;27(12):2938–47. doi: 10.1002/ejhf.70061 (PMC12803596; doi:10.1002/ejhf.70061)
Supplement: Supplementary file 1 — Appendix S1. Supporting Information. [file EJHF-27-2938-s001.docx]

**SUPPLEMENTARY MATERIAL**

**A U-shaped Relationship Between Left Ventricular Ejection Fraction and Risk of Worsening Heart Failure**

Hao-Chih Chang^a,b,c^, MD; Wei-Ming Huang^a,b,c^, MD, PhD; Liang-Yin Lin^d^, MS; Ching-Wei Lee^a^, MD; Chih-Hsueh Tseng^a,d,e^, MD; Wen-Chung Yu^a,b^, MD; Hao-Min Cheng^b,c,f^, MD, PhD; Chern-En Chiang^a,b,g^, MD, PhD; Chen-Huan Chen^b,c^, MD; Shih-Hsien Sung^a,b,d,g^*, MD, PhD.

^a^Division of Cardiology, Department of Medicine, Taipei Veterans General Hospital, Taipei, Taiwan;

^b^Cardiovascular Research Center, College of Medicine, National Yang Ming Chiao Tung University, Taipei, Taiwan;

^c^ Institute of Public Health, College of Medicine, National Yang Ming Chiao Tung University, Taipei, Taiwan;

^d^Institute of Emergency and Critical Care Medicine, College of Medicine, National Yang Ming Chiao Tung University, Taipei, Taiwan;

^e^Division of Holistic and Multidisciplinary Medicine, Department of Medicine, Taipei Veterans General Hospital, Taipei, Taiwan;

^f^ Division of Faculty Development, Department of Medical Education, Taipei Veterans General Hospital, Taipei, Taiwan;

^g^ General Clinical Research Center, Taipei Veterans General Hospital, Taipei, Taiwan.

*** Information for correspondence:**

Shih-Hsien Sung, M.D., Ph.D.

Institute of Emergency and Critical Care Medicine, National Yang Ming Chiao Tung University.

155, Sec. 2, Linong Street, Beitou District, Taipei 112304, Taiwan.

Tel: +886-2-2821-1699; Fax: +886-2-2820-2190.

E-mail: [mr.sungsh@gmail.com](mailto:mr.sungsh@gmail.com)

**Supplemental Methods.** ICD-9 and ICD-10 codes for comorbidities

| **Comorbidities*** | **ICD codes** | | |
| --- | --- | --- | --- |
| Hypertension | ICD-9 | | 401.x, 402.x, 403.x, 404.x, 405.x |
|  | ICD-10 | | I10–I13, I15–I16 |
| Diabetes | ICD-9 | 250.x | |
|  | ICD-10 | E11-E14 | |
| Hyperlipidemia | ICD-9 | 272.x | |
|  | ICD-10 | E78.x | |
| AF | ICD-9 | 427.31 | |
|  | ICD-10 | I48.0, I48.2, I48.91 | |
| CAD | ICD-9 | 414.xx | |
|  | ICD-10 | I25.x | |
| Heart failure | ICD-9 | 428.xx | |
|  | ICD-10 | I11.0, I13.0, I13.2, I50.x | |
| AF, atrial fibrillation; CAD, coronary artery disease; ICD, International Classification of Diseases. | | | |

* The presence of each comorbidity was defined by at least two independent outpatient diagnoses, the prescription of relevant medications, or documentation in the discharge diagnoses during hospitalization.

**Supplemental Figure S1.** Association between LVEF and the risk of the primary outcome, by treating LVEF as a time-varying variable.


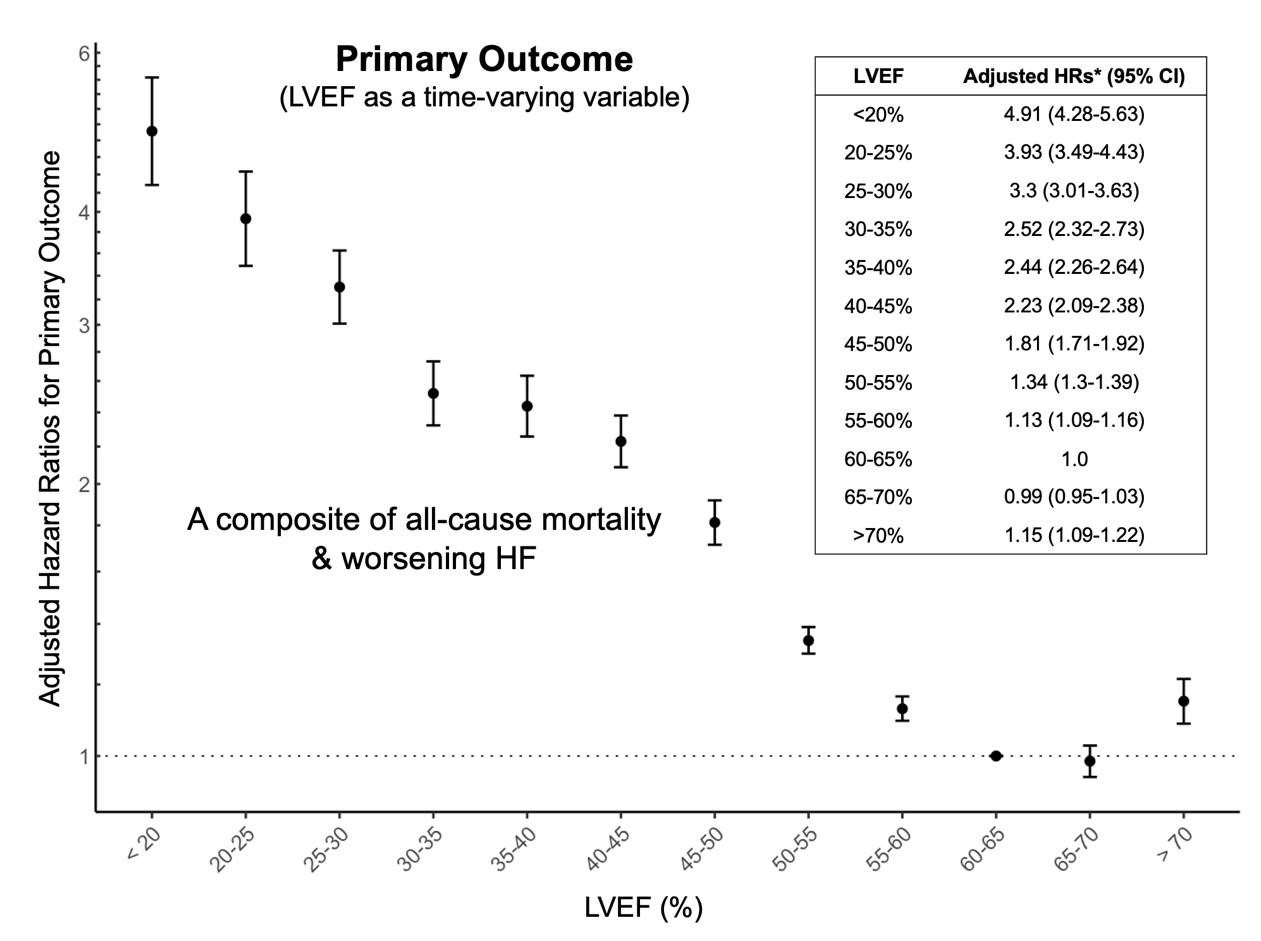


* Adjusting for age, sex, BMI, hypertension, diabetes, hyperlipidemia, atrial fibrillation, coronary artery disease, concurrent use of renin-angiotensin system inhibitors, β-blockers, mineralocorticoid receptor antagonists, and sodium-glucose cotransporter 2 inhibitors.

**Supplemental Figure S2.** Association between LVEF and the risk of secondary outcomes (A) all-cause mortality or cardiovascular death (B) worsening heart failure, by treating LVEF as a time-varying variable.

1. **All-cause mortality or cardiovascular death**


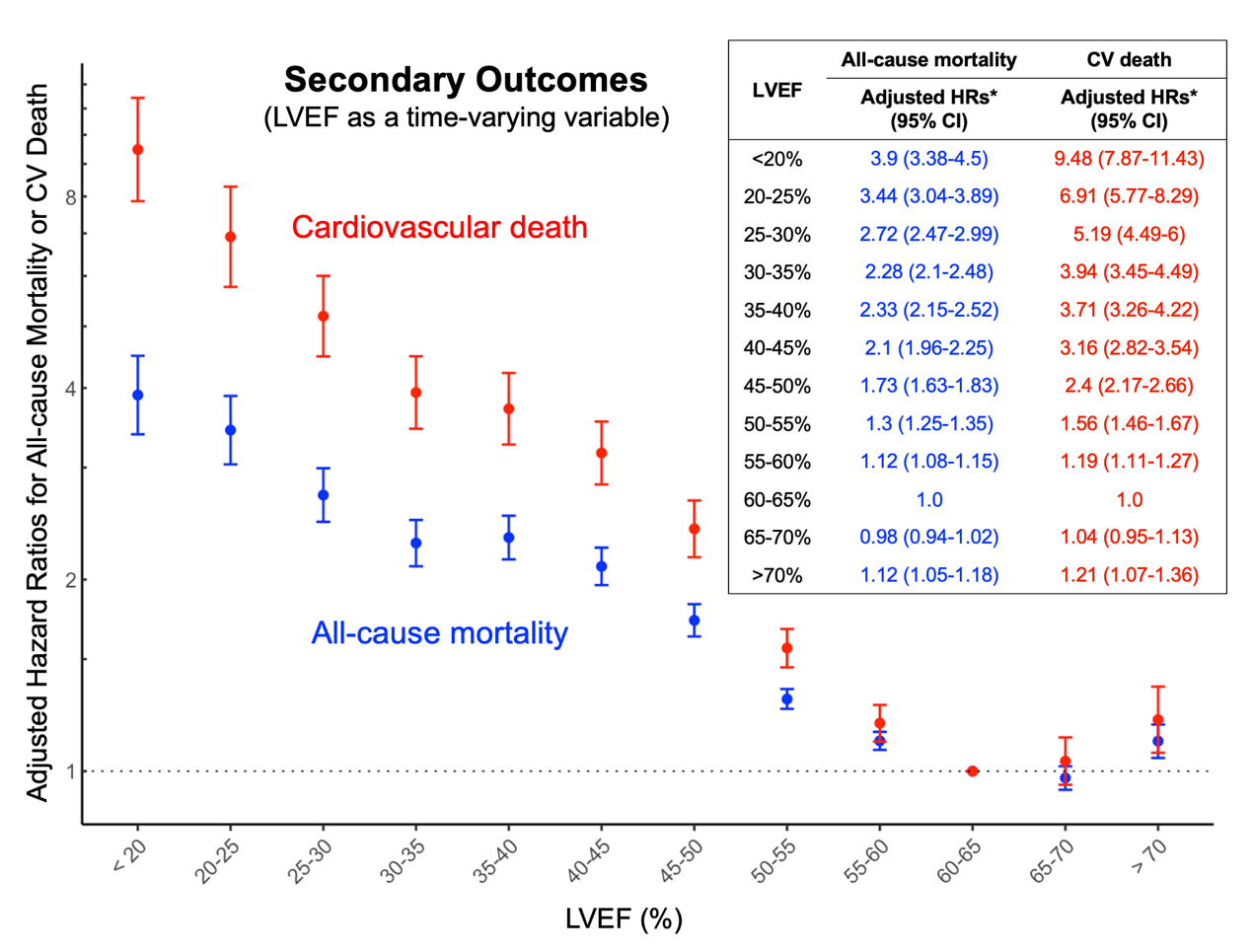


* Adjusting for age, sex, BMI, hypertension, diabetes, hyperlipidemia, atrial fibrillation, coronary artery disease, concurrent use of renin-angiotensin system inhibitors, β-blockers, mineralocorticoid receptor antagonists, and sodium-glucose cotransporter 2 inhibitors.

1. **Worsening heart failure**


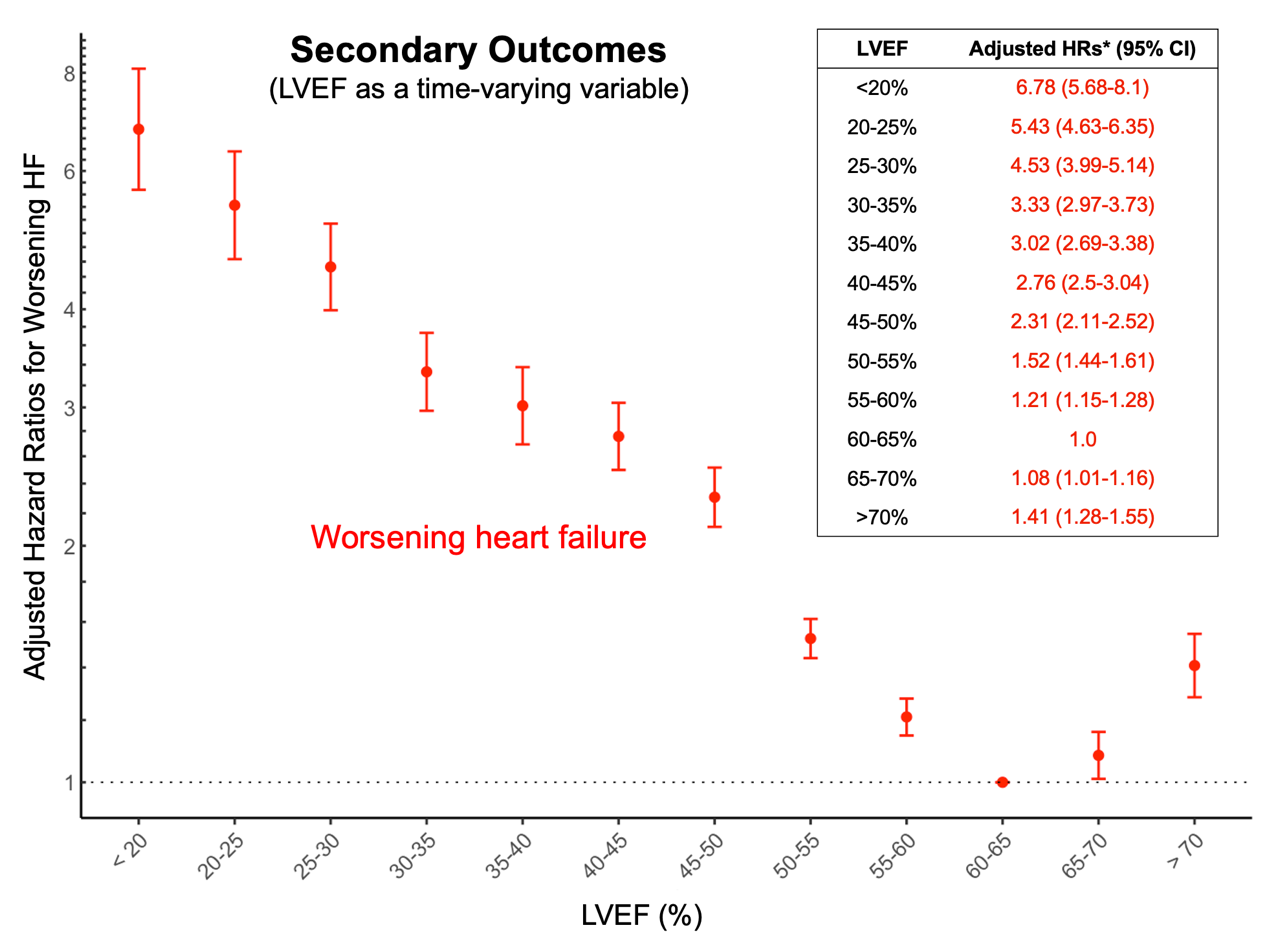


* Adjusting for age, sex, BMI, hypertension, diabetes, hyperlipidemia, atrial fibrillation, coronary artery disease, concurrent use of renin-angiotensin system inhibitors, β-blockers, mineralocorticoid receptor antagonists, and sodium-glucose cotransporter 2 inhibitors.

**Supplemental Figure S3.** Association between LVEF and the risk of HF hospitalization.


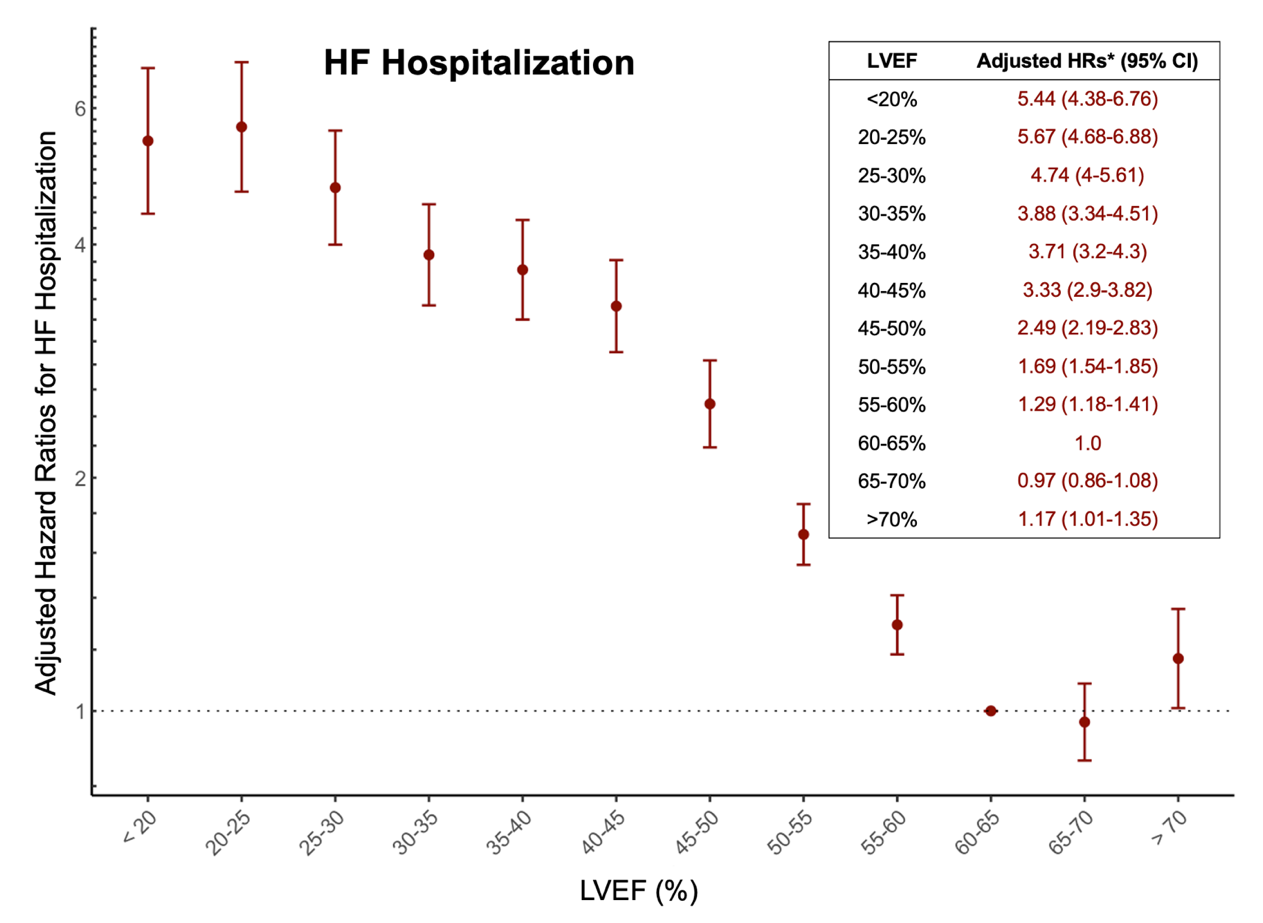


* Adjusting for age, sex, BMI, hypertension, diabetes, hyperlipidemia, atrial fibrillation, coronary artery disease, concurrent use of renin-angiotensin system inhibitors, β-blockers, mineralocorticoid receptor antagonists, and sodium-glucose cotransporter 2 inhibitors.

**Supplemental Figure S4.** Association between LVEF and the risk of incident HF, by excluding history of HF.


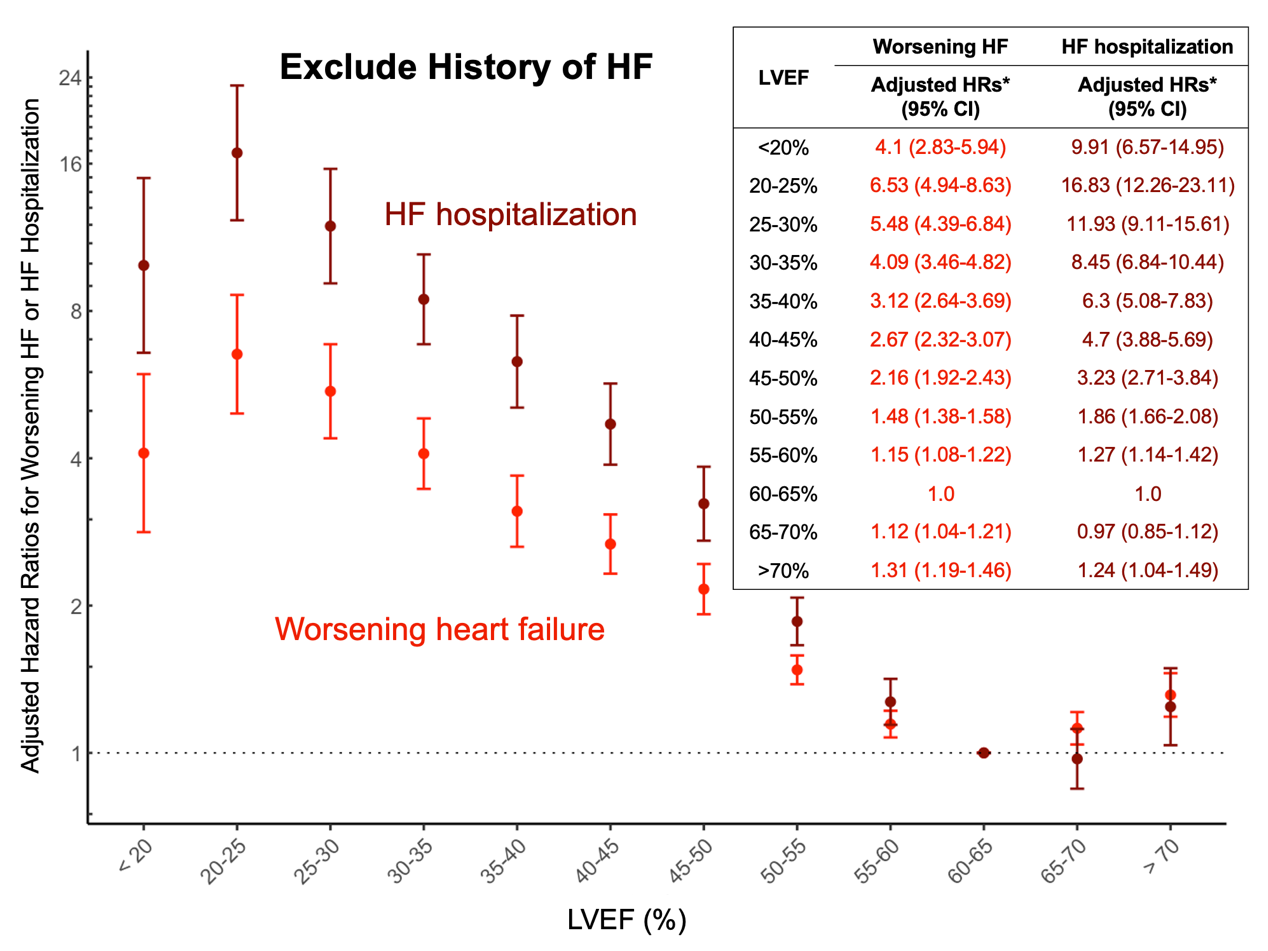


* Adjusting for age, sex, BMI, hypertension, diabetes, hyperlipidemia, atrial fibrillation, coronary artery disease, concurrent use of renin-angiotensin system inhibitors, β-blockers, mineralocorticoid receptor antagonists, and sodium-glucose cotransporter 2 inhibitors.

**Supplemental Figure S5.** Association between LVEF and the risk of incident HF, by excluding baseline use of oral loop diuretics.

**
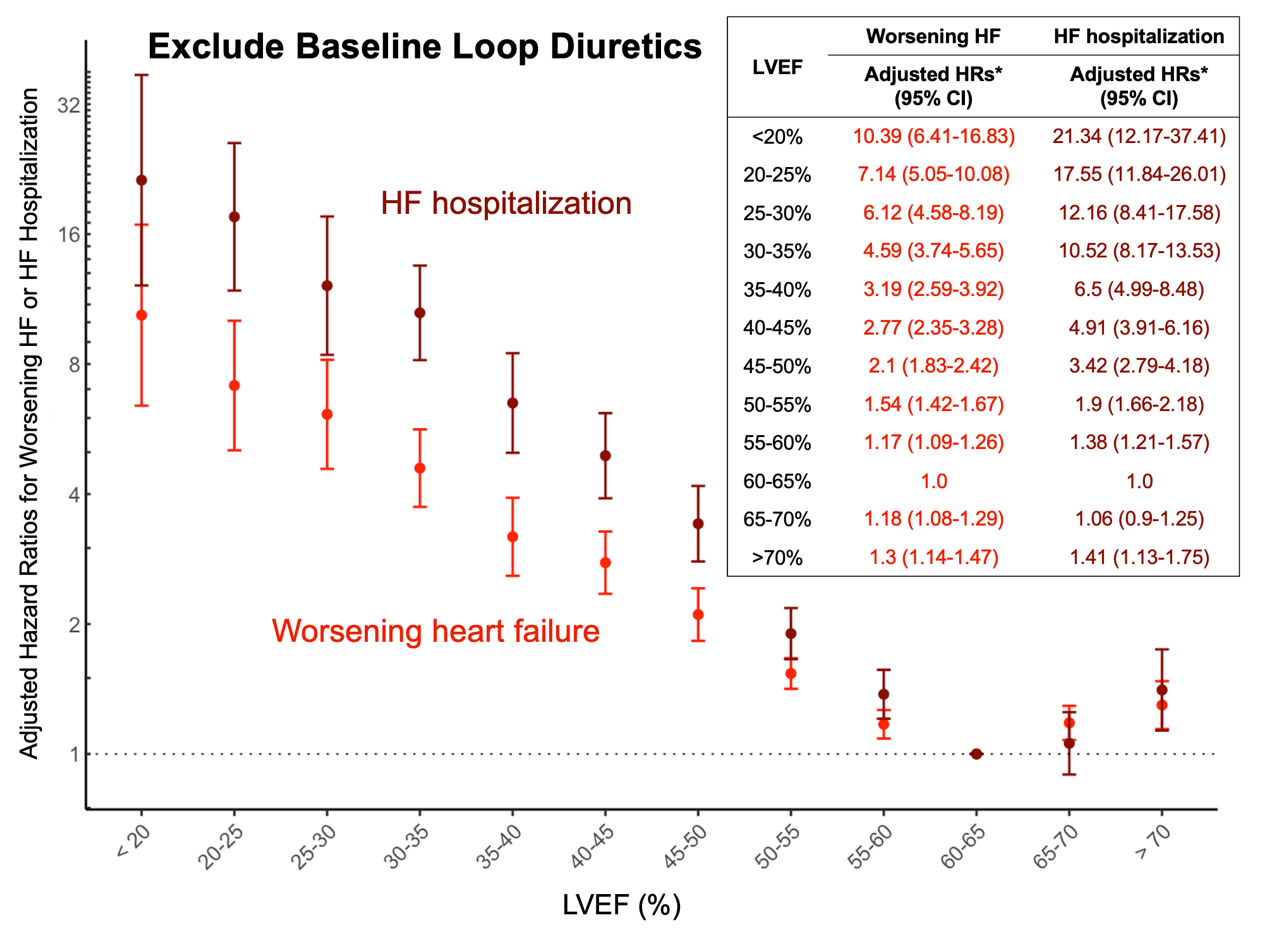
**

* Adjusting for age, sex, BMI, hypertension, diabetes, hyperlipidemia, atrial fibrillation, coronary artery disease, concurrent use of renin-angiotensin system inhibitors, β-blockers, mineralocorticoid receptor antagonists, and sodium-glucose cotransporter 2 inhibitors.

**Supplementary, Figure S6** Association between LVEF and the risk of recurrent HF hospitalization.


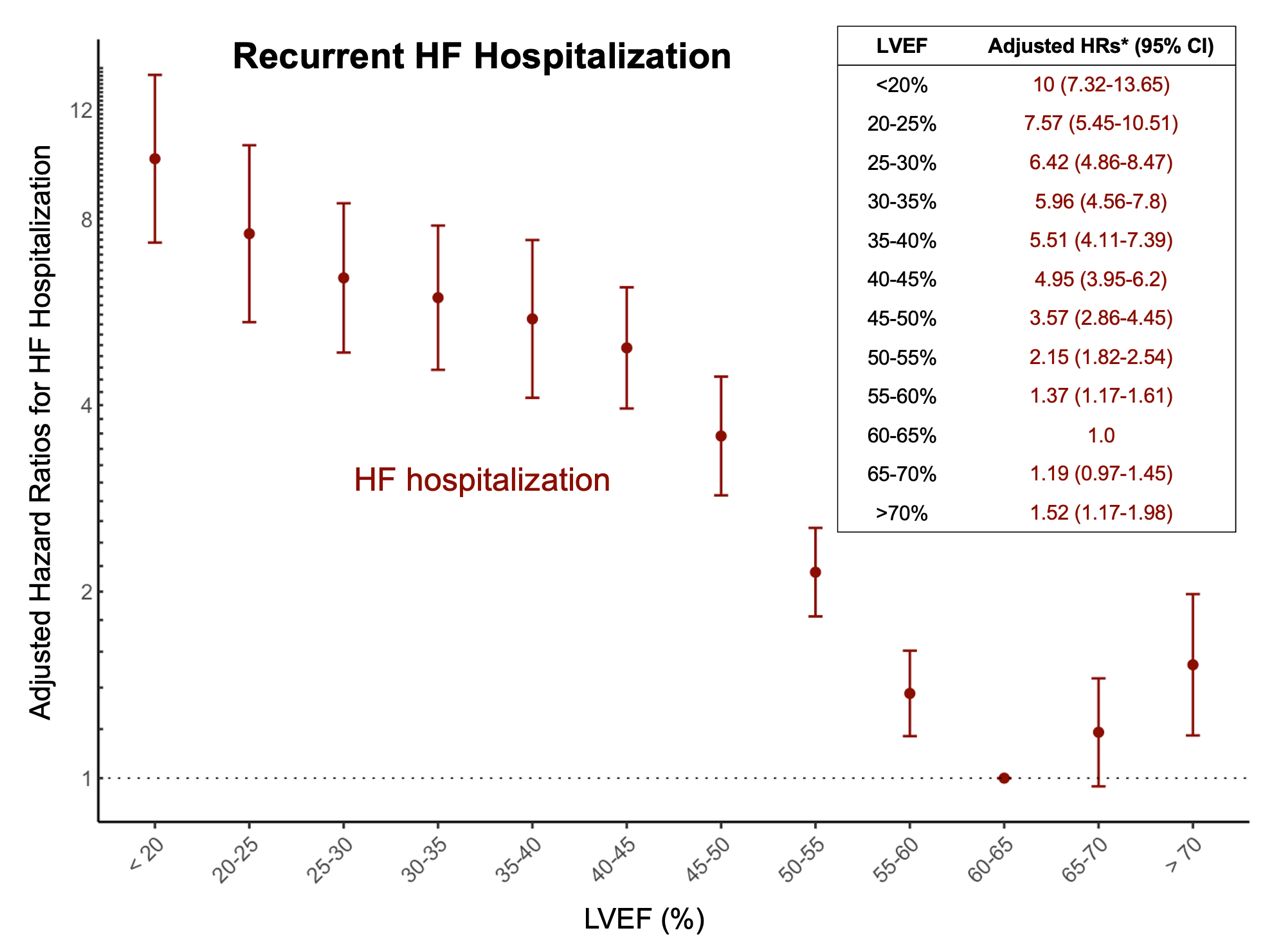


* Adjusting for age, sex, BMI, hypertension, diabetes, hyperlipidemia, atrial fibrillation, coronary artery disease, concurrent use of renin-angiotensin system inhibitors, β-blockers, mineralocorticoid receptor antagonists, and sodium-glucose cotransporter 2 inhibitors.

**Supplementary Figure S7** Association between LVEF and the risk of primary outcome, with further adjustment for indications of echocardiography.


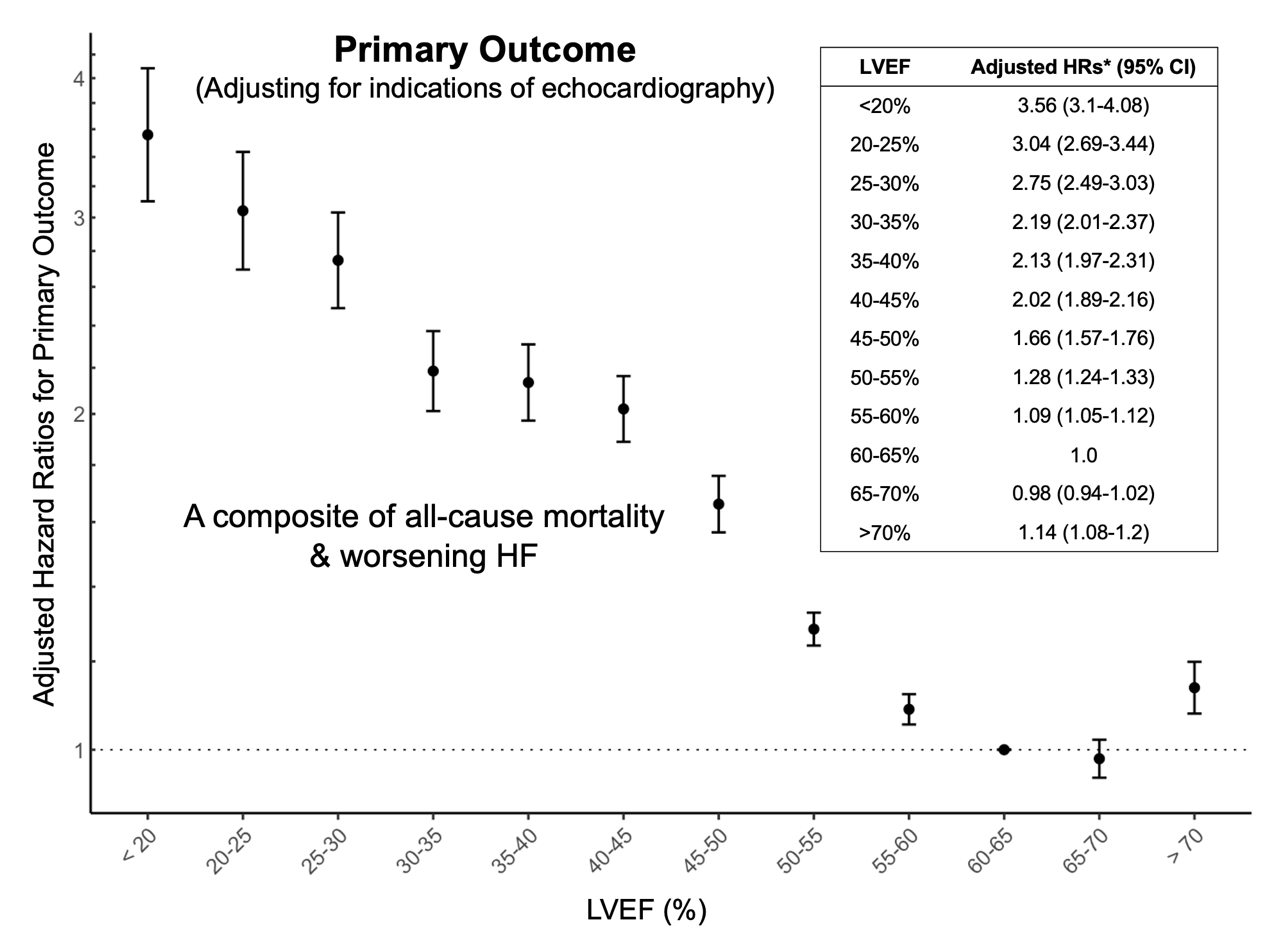


* Adjusting for age, sex, BMI, hypertension, diabetes, hyperlipidemia, atrial fibrillation, coronary artery disease, concurrent use of renin-angiotensin system inhibitors, β-blockers, mineralocorticoid receptor antagonists, sodium-glucose cotransporter 2 inhibitors, and indications of echocardiography.

**Supplemental Figure S8.** Association between LVEF and the risk of secondary outcomes (A) all-cause mortality or cardiovascular death (B) worsening heart failure, with adjustment for indications of echocardiography.

1. **All-cause mortality or cardiovascular death**


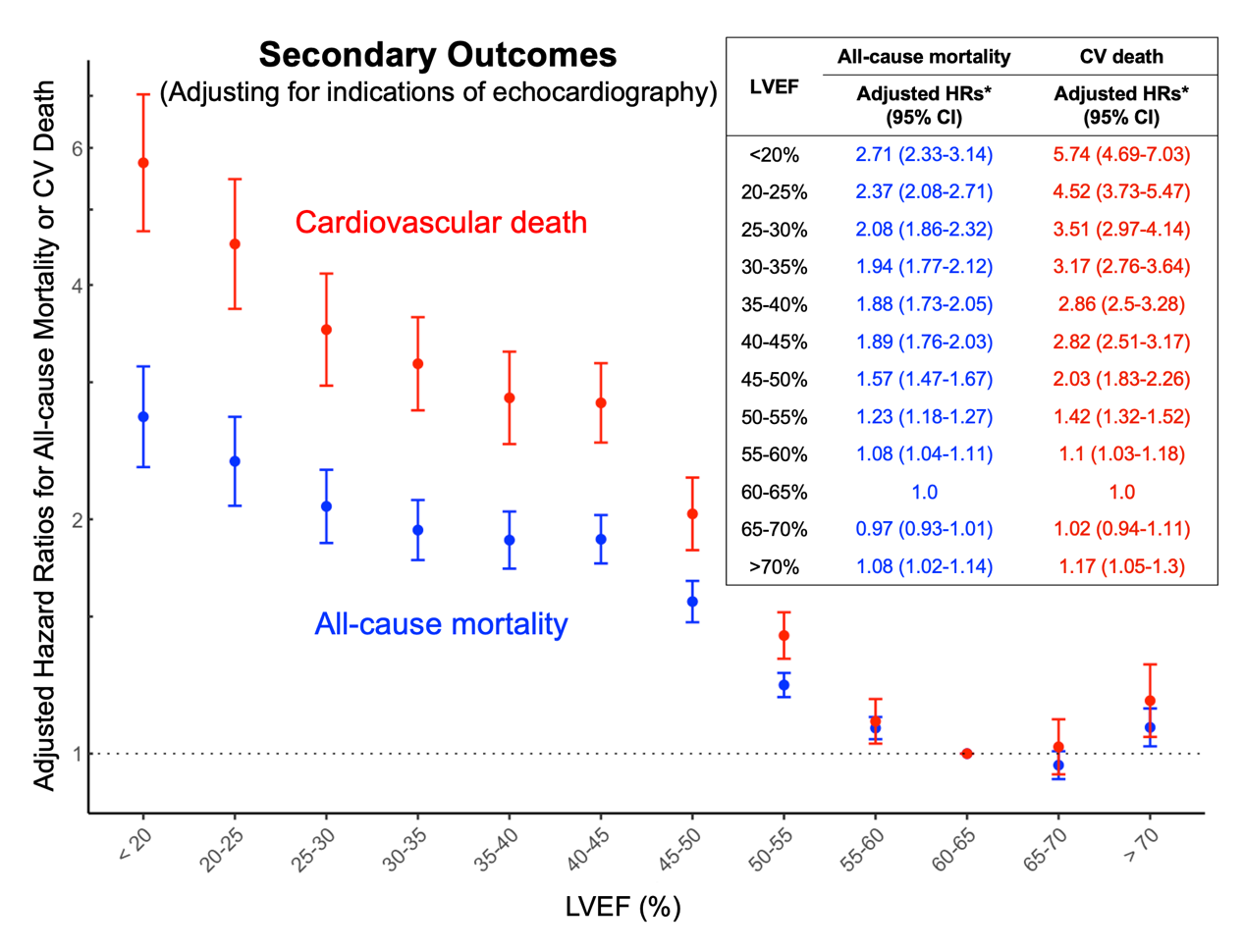


* Adjusting for age, sex, BMI, hypertension, diabetes, hyperlipidemia, atrial fibrillation, coronary artery disease, concurrent use of renin-angiotensin system inhibitors, β-blockers, mineralocorticoid receptor antagonists, sodium-glucose cotransporter 2 inhibitors, and indications of echocardiography.

1. **Worsening heart failure**


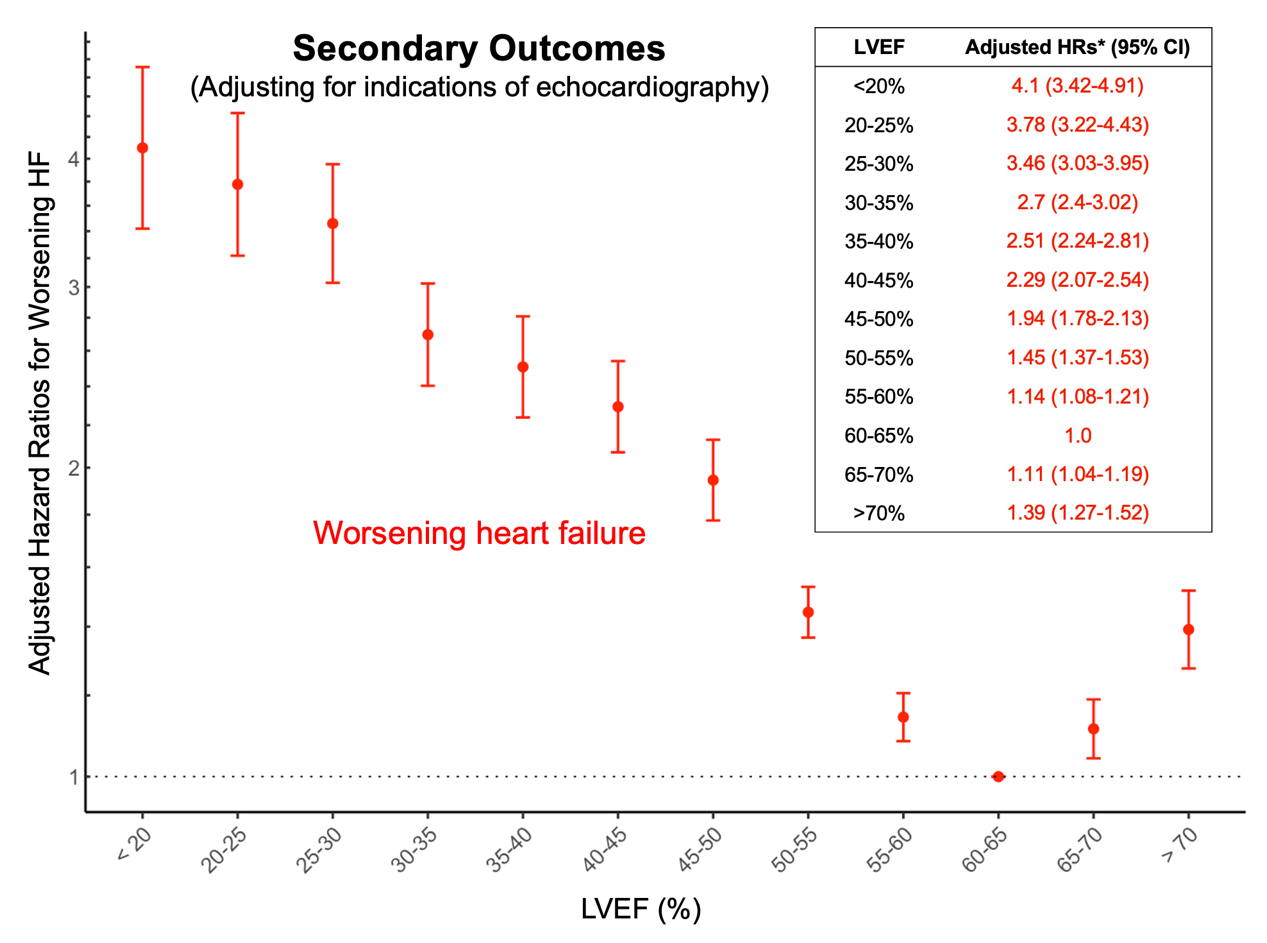


* Adjusting for age, sex, BMI, hypertension, diabetes, hyperlipidemia, atrial fibrillation, coronary artery disease, concurrent use of renin-angiotensin system inhibitors, β-blockers, mineralocorticoid receptor antagonists, sodium-glucose cotransporter 2 inhibitors, and indications of echocardiography.

**Supplemental Figure S9.** Association between LVEF and the risk of worsening heart failure by subgroups of age or sex.

1. **Age subgroups**


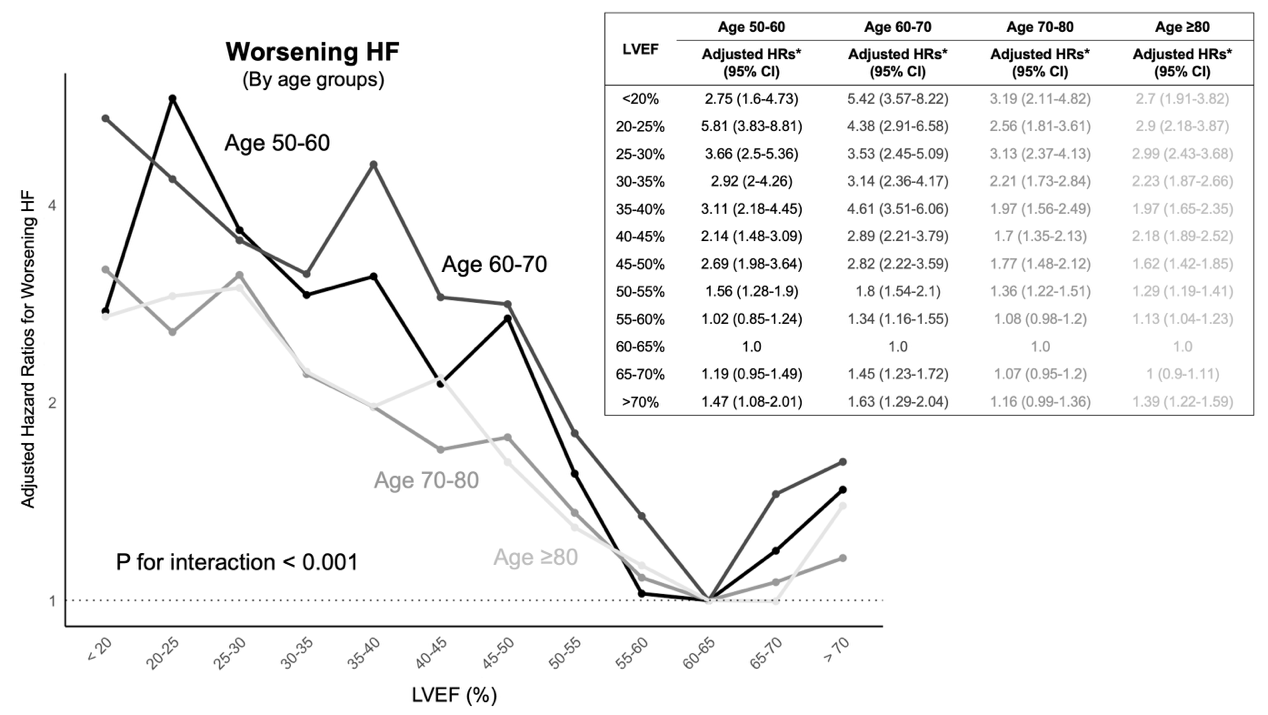


* Adjusting for sex, BMI, hypertension, diabetes, hyperlipidemia, atrial fibrillation, coronary artery disease, concurrent use of renin-angiotensin system inhibitors, β-blockers, mineralocorticoid receptor antagonists, and sodium-glucose cotransporter 2 inhibitors.

1. **Sex**


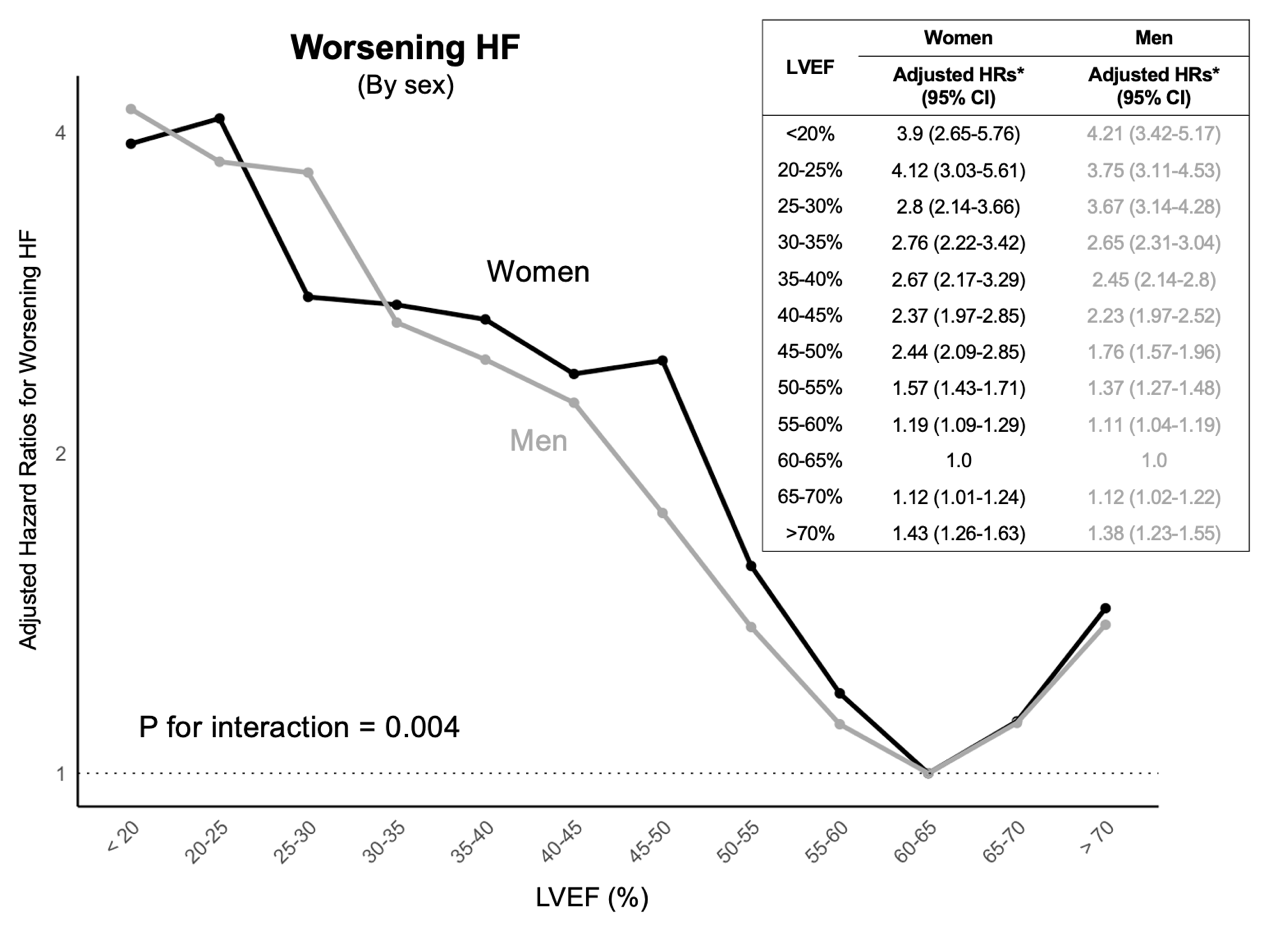
* Adjusting for age, BMI, hypertension, diabetes, hyperlipidemia, atrial fibrillation, coronary artery disease, concurrent use of renin-angiotensin system inhibitors, β-blockers, mineralocorticoid receptor antagonists, and sodium-glucose cotransporter 2 inhibitors.

**Supplemental Figure S10.** Association between LVEF and the risk of worsening heart failure by subgroups of hypertension or diabetes.

1. **Hypertension**

**
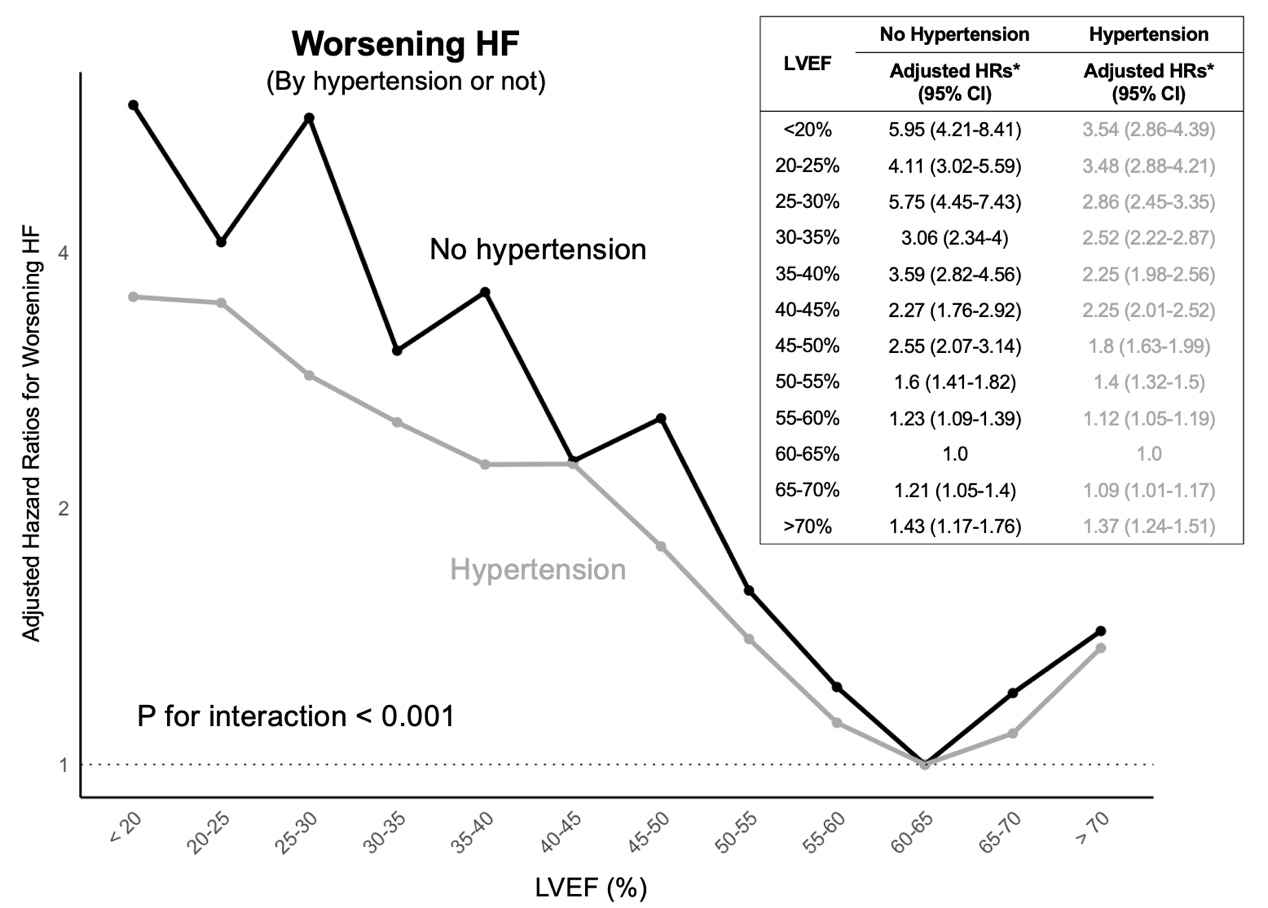
**

* Adjusting for age, sex, BMI, diabetes, hyperlipidemia, atrial fibrillation, coronary artery disease, concurrent use of renin-angiotensin system inhibitors, β-blockers, mineralocorticoid receptor antagonists, and sodium-glucose cotransporter 2 inhibitors.

1. **Diabetes**

**
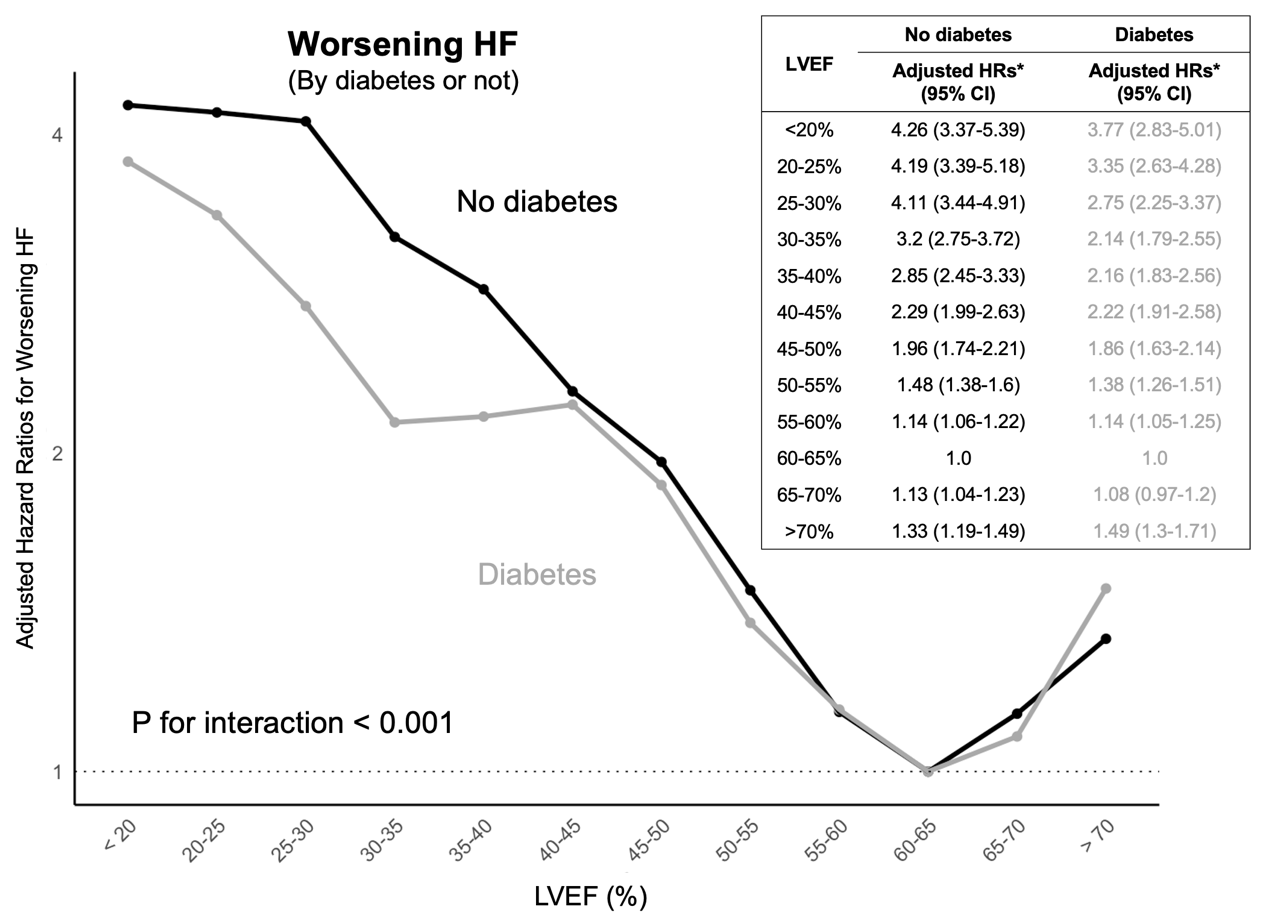
**

* Adjusting for age, sex, BMI, hypertension, hyperlipidemia, atrial fibrillation, coronary artery disease, concurrent use of renin-angiotensin system inhibitors, β-blockers, mineralocorticoid receptor antagonists, and sodium-glucose cotransporter 2 inhibitors.
